# Evaluation of Synaptic and Axonal Dysfunction Biomarkers in Alzheimer’s Disease and Mild Cognitive Impairment Based on CSF and Bioinformatic Analysis

**DOI:** 10.3390/ijms231810867

**Published:** 2022-09-17

**Authors:** Maciej Dulewicz, Agnieszka Kulczyńska-Przybik, Renata Borawska, Agnieszka Słowik, Barbara Mroczko

**Affiliations:** 1 Department of Neurodegeneration Diagnostics, Medical University of Bialystok, 15-269 Bialystok, Poland; 2 Department of Neurology, Jagiellonian University, 30-688 Kraków, Poland; 3 Department of Biochemical Diagnostics, Medical University of Bialystok, 15-269 Bialystok, Poland

**Keywords:** neurogranin, neuronal pentraxin receptor, Visinin-like protein 1, CSF synaptic biomarkers

## Abstract

Synaptic loss and dysfunction are one of the earliest signs of neurodegeneration associated with cognitive decline in Alzheimer’s disease (AD) and other neurodegenerative diseases. This study aimed to assess the relationships between biological processes of the synaptic pathology underlying AD, molecular functions, and dynamics of the change concentrations of selected proteins reflecting synaptic and axonal pathology in dementia stages. Neurogranin (Ng), neuronal pentraxin receptor (NPTXR), and Visinin-like protein 1 (VILIP1) concentrations were measured in the cerebrospinal fluid (CSF) of MCI, AD, and non-demented controls (CTRL) using quantitative immunological methods. Gene ontology (GO) enrichment analysis was used for the functional analysis of tested proteins. The CSF Aβ42/Ng ratio was significantly different between all the compared groups. The CSF NPTXR/Ng ratio was significantly different between MCI compared to CTRL and AD compared to CTRL. The GO enrichment analysis revealed that two terms (the Biological Process (BP) and Cellular Component (CC) levels) are significantly enriched for NPTXR and Ng but not for VILIP1. Both Ng and NPTXR concentrations in CSF are promising synaptic dysfunction biomarkers for the early diagnosis of the disease. Moreover, both proteins are biochemically associated with classical biomarkers and VILIP-1. Mapping shared molecular and biological functions for the tested proteins by GO enrichment analysis may be beneficial in screening and setting new research targets.

## 1. Introduction

Alzheimer’s Disease (AD) is the leading cause of dementia [1,2]. The etiology and early pathogenesis of AD are still unclear. AD’s most common neuropathological changes include extracellular depositions of amyloid-beta peptides, especially Aβ1–42, and intracellular neurofibrillary tangles (NFT) composed of hyperphosphorylated Tau [2]. The classical biomarkers widely studied and used in clinical practice are the proteins Aβ1–42, total Tau (t-tau), and pTau181. These three CSF biomarkers were included for AD diagnosis established by The National Institute of Aging and Alzheimer’s Association (NIA-AA) guidelines and International Work Group (IWG) [3]. One of the first symptoms is progressive cognitive decline related to Aβ deposits, neurofibrillary tangles, and synapse loss in crucial brain regions, such as the hippocampus. Mental disability, including memory disturbance, is the earliest symptom of AD. Memory processes are generally associated with hippocampal function and neuronal communication maintained by synapses. The impairment of neuronal transmission between synapses is associated with early neurodegenerative changes and cognitive deficits. Some mechanisms leading to synaptic dysfunction are observed and described in neurodegenerative diseases [4].

In this study, we decided to investigate three proteins: neurogranin (Ng), neuronal pentraxin receptor (NPTXR), and Visinin-like protein 1 (VILIP-1), related to synaptic plasticity or calcium signaling. Neurogranin is a small synaptic protein that influences the induction of LTP by binding to calmodulin (CaM) in response to low Ca2+ levels [5] Other studies suggest that Ng is involved in LTP via Ca2+ and CaM signaling pathways, essential for synaptic plasticity and regeneration [3,6] . In contrast, NPTXR is a unique transmembrane protein belonging to the neuronal pentraxin family [7] The highest expression of NPTXR and involvement in neuronal processes have been observed in the hippocampus and neocortex [7,8]. It has been suggested that NPTXR affects synapse formation and is also responsible for synaptic transmission by attaching to AMPARs [7]. The VILIP1 is a neuronal calcium sensor protein associated with calcium signaling and interaction with α4β2 nAChR [9]. However, VILIP1 has been described as a modulator of cell-surface-associated protein, especially with membranes of axons and dendrites [10]. Reduced levels of nAChRs and cholinergic neurotransmission have been implicated in the etiology of AD, and acetylcholinesterase inhibitors are used to treat AD [10,11] Given these reports on the critical role of proteins modulating synaptic plasticity in the pathogenesis of AD, it seems reasonable to investigate their potential clinical utility and compare them with classical biomarkers. We also performed preliminary bioinformatic analysis to assess the possible relationships between biological processes and tested proteins.

## 2. Results

### 2.1. Bioinformatic Analyses and Mapping of Possible Pathways between Tested Proteins and Alzheimer’s Disease

**Figure 1:**
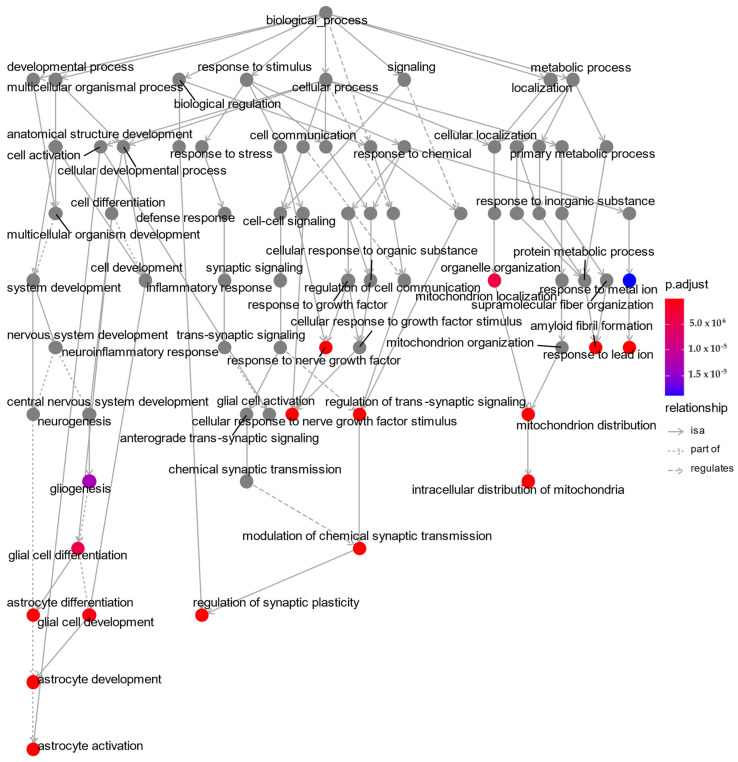
GO plot biological processes with dependencies between them based on enriched gene ontology terms for MAPT, APP, NRGN, and NPTXR. Top 10 biological processes were highlighted as color dots. This plot was produced in ClusterProfiler; p.adjust  =  the Benjamini–Hochberg adjusted *p*-value for the enriched ontology term.

The specific terms of Gene Ontology (GO) analysis are widely used for the discovery and understanding of the biological roles of target proteins in three categories, namely, cellular component (CC), molecular function (MF), and arrangement of biological processes (BP). Additionally, GO term enrichment analysis provides functional interpretations of targeted proteins based on sets of genes and associated terms of hierarchically classified categories. In our research, we decided to use the gene names of coding proteins examined in CSF for performing preliminary, and screening GO analysis. The results of the Go enrichment analysis shown in Table 1 and Figure 1 were created based on the following input gene names: MAPT, APP, NRGN, and NPTXR. The corresponding gene names were representations of the tested proteins as follows: MAPT = Tau protein, APP = amyloid precursor protein, NRGN = neurogranin, NPTXR = neuronal pentraxin receptor. The top 10 BP terms enriched with four genes are presented in the hierarchical GO plot (Figure 1) with all related biological processes. We chose the five proteins (Ng, NPTXR, VILIP-1, Tau, and Aβ42) and examined them using an over-representation test, which revealed that four genes (MAPT, APP, NRGN, NPTXR) are involved in GO terms for biological processes, including GO:0050804—“modulation of chemical synaptic transmission” and GO:0099177—“regulation of trans-synaptic signaling” (Table 1). However, for GO cellular component terms, significant enrichment analysis was found only for MAPT, APP, and NGRN genes related to GO:0043197—“dendritic spine”, GO:0044309—“neuron spine”, and GO:0043025—“neuronal cell body”, respectively.

### 2.2. Candidates’ Biomarkers Concentrations in Cerebrospinal Fluid

**Figure 2:**
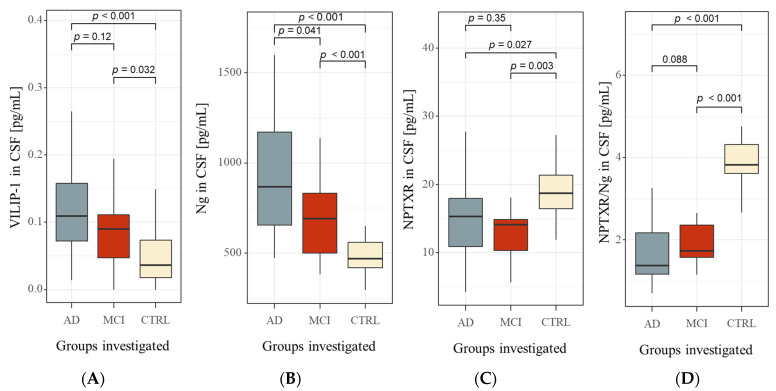
Boxplots of CSF concentrations of tested biomarkers (**A**) VILIP-1, (**B**) Ng, (**C**) NPTXR, and (**D**) NPTXR/Ng in examined groups. Abbreviations: cerebrospinal fluid (CSF), Visinin-like protein 1 (VILIP1), neurogranin (Ng), neuronal pentraxin receptor (NPTXR), neuronal pentraxin receptor/neurogranin ratio, Alzheimer’s disease (AD), mild cognitive impairments (MCI), control group (CTRL).

The concentrations of NPTXR, Ng, and VILIP-1 and calculated ratios (Aβ42/Ng and Ng/NPTXR) in the cerebrospinal fluid are shown in the first table (Table 2). Table 2 also shows the biochemical characteristics of novel biomarkers ratios, such as the Aβ42/Ng ratio (*p* < 0.001) and Ng/NPTXR (*p* < 0.001). Based on the Kruskal–Wallis test, the significant differences in all tested groups were observed for CSF levels of the Aβ42/40 ratio (*p* < 0.001), Aβ42 (*p* < 0.001), Tau (*p* < 0.001), pTau181 (*p* < 0.001), NPTXR (*p* < 0.001), Ng (*p* < 0.001), and VILIP-1 (*p* < 0.001). The post hoc Dwass–Steele–Critchlow–Fligner test revealed that the Ng levels in CSF differed significantly between tested groups of patients and the CTRL group (Table 2, Figure 2B). The CSF NPTXR levels were significantly higher in AD and MCI patients compared to the CTRL, although the difference was not significant between MCI and AD groups. The levels of VILIP1 have a similar trend as NPTXR without statistically significant differences between AD and MCI (Table 2, Figure 2A). Additionally, there were no significant differences between MCI and CTRL groups (Figure 2A).

### 2.3. Associations between CSF Levels of Ng, NPTXR, and VILIP1 and Neurochemical Biomarkers (Aβ42/40 Ratio, Tau, and pTau181)

The associations between levels of Ng, NPTXR, and VILIP-1 and neurochemical biomarkers were performed using the Spearman rank correlation test. Significantly positive correlations were observed in the whole study group (AD + MCI + CTRL) between CSF Ng and VILIP-1 (rho = 0.646, *p* < 0.001), age (rho = 0.340, *p* = 0.004), Tau (rho = 0.728, *p* < 0.001), and pTau181 (rho = 0.749, *p* < 0.001) and negative with MMSE (rho = −0.438, *p* < 0.001) and the Aβ42/40 ratio (rho = −0.365, *p* < 0.01). Positive correlations were observed between NPTXR and VILIP1 (rho = 0.249, *p* = 0.037) and negative with Aβ42 (rho = −0.438, *p* < 0.001). The CSF levels of VILIP-1 were positively correlated with age (rho = 0.308, *p* = 0.009) and Tau (rho = 0.706, *p* < 0.001) and negatively correlated with MMSE (rho = −0.410, *p* < 0.001) and the Aβ42/40 ratio (rho = −0.446, *p* < 0.001).

In the AD group, the CSF levels of Ng significantly correlated with the concentration of VILIP-1 (rho = 0.646, *p* < 0.001), age (rho = 0.340, *p* = 0.004), Tau (rho = 0.728, *p* < 0.001), pTau181 (rho = 0.749, *p* < 0.001), and NPTXR (rho = −0.181, *p* = 0.040). The NPTXR in CSF positively correlated with VILIP-1 (rho = 0.500, *p* = 0.003), Tau (rho = 0.506, *p* = 0.003), and pTau181 (rho = 0.574, *p* < 0.001). VILIP1 positively correlated with Aβ42 (rho = 0.397, *p* = 0.022), Tau (rho = 0.650, *p* < 0.001), and pTau181 (rho = 0.673, *p* < 0.001).

In the MCI group, CSF levels of Ng significantly positively correlated with NPTXR (rho = 0.799, *p* < 0.001), VILIP1 (rho = 0.598, *p* = 0.009), Aβ42 (rho = 0.748, *p* < 0.001), Tau (rho = 0.680, *p* = 0.003), and pTau181 (rho = 0.667, *p* = 0.003). The CSF NPTXR positively correlated with Tau (rho = 0.680, *p* = 0.003) and pTau181 (rho = 0.668, *p* = 0.003).

### 2.4. Diagnostic Usefulness of Candidate Biomarkers and Ratios

**Figure 3:**
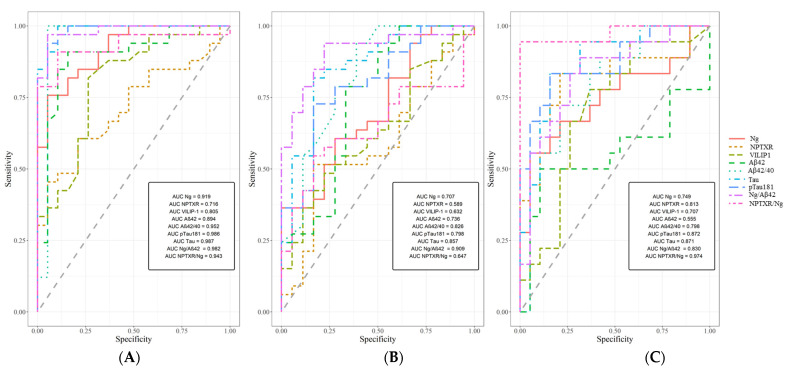
Areas under ROC curves (AUC) for CSF Ng, NPTXR, VILIP-1, Aβ42/Ng, Ng/NPTXR, and classical biomarkers in (**A**) AD compared to CTRL; (**B**) AD compared to MCI; (**C**) MCI compared to CTRL. Ng—neurogranin, NPTXR—neuronal pentraxin receptor, VILIP-1—Visinin-like protein 1, Aβ—amyloid beta, Aβ42/40—ratio of amyloid beta 1-42 and 1-40, Aβ42/Ng—ratio of amyloid beta 42 and neurogranin, AD—Alzheimer’s disease, CTRL—controls, MCI—mild cognitive impairment.

An analysis of the receiver operating characteristic curve (ROC) showed that the CSF levels of neurogranin may significantly discriminate AD patients from controls (AUC = 0.919, 95% CI 78.4–99.55, *p* < 0.001), with 81% accuracy, 82% specificity, and 79% sensitivity. The NPTXR levels may significantly differentiate AD patients from controls (AUC = 0.751, *p* = 0.001), with 68% accuracy, 80% specificity and 62% sensitivity. The AUC analysis of VILIP-1 was statistically significant (AUC = 0.805, *p* < 0.001), with 77% accuracy, 79% specificity, and 74% sensitivity. The AUCs for all tested proteins and classical biomarkers are presented in Figure 3 and Table 3. The AUCs of the candidate’s biomarkers and ratios were compared to classical biomarkers via DeLong’s test. The comparison analysis in the MCI versus CTRL groups showed a significant difference between NPTXR/Ng and Aβ42/40 ratios (AUC differences = 0.173 [0.022–0.323], *p* = 0.025). An analysis of ROC also compared MCI and AD patients, where the Aβ42/Ng ratio had the highest AUC value. The significant results of the ROC are presented in Figure 3 and Table 3.

## 3. Discussion

The main objective of this study was to evaluate the associations between biological processes of the synaptic pathology underlying this disease, the molecular functions of some causative proteins, and the dynamics of the change in concentrations of selected proteins reflecting synaptic and axonal pathology (Ng, NPTXR, VILIP1, the NPTXR/Ng ratio, and the Aβ42/Ng ratio) in dementia stages. We used a bioinformatics approach to establish the functions of proteins using GO enrichment analysis. By applying bioinformatics tools to experimental data, we can better understand and interpret the results of the biological functions of tested proteins. Enrichment analyses, such as GO, DO, and KEGG, are widely used for high-throughput experiments (e.g., RNA seq) or determining which GO terms appear more frequently in a set of genes [12]. This analysis technique was used in our study to see which biological processes might correspond to defined proteins based on their gene names.

The loss of synapses seems to be very close to Aβ plaque formation. The exact pathway of impact and role of Aβ are still being researched. One of the possible pathways of impact is related to Aβ-triggered Ca2+ influx and induced calcium dyshomeostasis in the endoplasmic reticulum (ER), mitochondrion, and whole neurons [13,14]. The altered Ca2+ homeostasis by Aβ may cause excitotoxicity and neuronal death [14]. The second possible pathway is related to synaptic transmission and plasticity, as the crucial processes of memory depend on long-term potentiation (LTP) and long-term depression (LTD) [15]. Aβ, through its ability to bind to N-methyl-D-aspartate receptors (NMDARs), α-amino-3-hydroxy-5-methyl-4-isoxazolopropionate receptors (AMPARs), and nicotinic acetylcholine receptors, makes them permeable for Ca2+ [5,6]. Aβ oligomers (Aβo) mainly accumulate at the excitatory synaptic sites of glutamatergic neurons, deregulate NMDA signaling pathways, and inhibit long-term potentiation [6,16,17]. The synergistic mechanism of Aβ and Ca2+ could promote neurodegeneration and cognitive deficits in AD and MCI patients [14]. In the glutamatergic synapses, Ca2+ influx through LTP activates calcium-calmodulin-dependent protein kinase II (CaMKII) depending on the availability of calmodulin (CaM) [18]. The availability of CaM depends on neurogranin (Ng) [19,20]. Ca2+ alters the affinity of calmodulin and, upon activation of the CaMKII, interacts with neurotransmitter receptors inside the synapse [19]. CaMKII interactions play a crucial role in strengthening synapses [19]. However, altered calcium signaling may also be associated with the expression or response of calcium-binding and sensing proteins [13,14]. Research focused on synaptic proteins can help us to better understand neurobiological mechanisms related to dysfunctions of memory, one of the earlier signs of AD [4,14,21]. Several mechanisms and pathways regulate the pathological dysregulation of synaptic transmission and other conditions in AD. Therefore, panels of proteins should be used to better understand the pathological conditions in neurodegenerative diseases.

In our study, we performed a bioinformatic analysis and combined it with an assessment of the concentrations of synaptic dysfunction biomarkers (Ng, NPTXR), as well as one for neuronal injury (VILIP1), to verify the association between the analysis of molecular functions and dynamics of the concentration changes in dementia stages. An enrichment analysis based on gene names was performed to find out more precisely in which biological processes all proteins might be involved. Enrichment analysis was intended to point to common pathways and cellular components, even though our study is not a genomic study [12]. Enrichment analysis revealed several important processes in which the selected proteins are involved. Two processes proved particularly important for all the tested proteins and their corresponding gene names: the modulation of chemical synaptic transmission and the regulation of trans-synaptic signaling. In contrast, more relationships are shared between NGRN and APP: “positive regulation of long-term synaptic potentiation”, “regulation of long-term synaptic potentiation, associative learning”, “long-term synaptic potentiation”, “learning”, and “positive regulation of synaptic transmission”. All of the above processes appear to be particularly relevant in early signs of AD and justify using the Aβ42/Ng ratio. Interestingly, “astrocyte activation” also proved to be significant, which is essential for the release of glutamate and the cascade of pathological processes. This study showed that this type of bioinformatic analysis could be applied even in a very narrow scope. It is likely that the use of more genes encoding relevant proteins in AD could give more extensive results. In addition, bioinformatics analysis provides a better understanding of which proteins are involved in biological processes, in which regions of the brain, and in which cell types they are highly expressed. However, experimental data should be carried out on a larger cohort, and bioinformatic analysis should be replicated by other researchers with the same background genes.

The levels of Ng increased progressively from MCI to AD compared to CTRL. Our results confirm the general trend associated with increased CSF Ng levels concerning disease progression [22]. Interestingly, the increase in Ng concentration may be related to the loss of glutamatergic synapses, one of the key and early signs of memory problems [19,23]. The accurate diagnosis of early changes before the MCI stage seems to be a particularly crucial diagnostic goal. The NPTXR, also an important molecule for glutamatergic synaptic transmission, similarly to Ng, not only proved to be statistically significant in the MCI group but also in AD patients compared to the CTRL group. Our results are in agreement with other studies [23,24]. The reduced NPTXR levels in AD and MCI groups may indicate early and persistent changes in the availability of glutamine and synapse reduction. Interestingly, we did not observe statistically significant differences in NPTXR levels between AD and MCI patients. The lack of differentiation between later stages of the disease may be due to very early changes in excitatory and inhibitory postsynaptic sites, especially in glutamatergic neurons or dyshomeostasis glutamate between synaptic cleft [7]. This is likely influenced by many overlapping processes rather than one that is strictly isolated. Nevertheless, NPTXR seems particularly relevant in the early stages of the disease but not in conversion from MCI to AD [24,25].

The correlations in the AD group, especially between Ng and Tau proteins (tTau and pTau181), may be related to synaptic loss and microtubule dysfunctions [26,27,28]. This relationship can be interpreted as reflecting cognitive decline, atrophy of the brain, and calcium dyshomeostasis [29]. Additionally, the positive correlation of the Ng with VILIP1 may reflect the involvement of both proteins in calcium signaling. Interestingly, both proteins influence calcium pathology by different receptors. On the one hand, Ng is strongly involved in Ca2+ signaling for NMDAR channels. On the other hand, VILIP1, as a neuronal Ca2+ sensor protein, may interact with the nicotinic acetylcholine receptor (nAChR) [10,11,30]. The arrangement of both receptors and proteins in memory and cognition dysfunction in AD and MCI pathology may be one of the important early pathological mechanisms. However, whether there is the involvement of multiple mental processes, or one mechanism of their joint action is still unclear.

The correlation between Ng and Aβ42 in the MCI group may be related to shrinkage of dendritic spines and glutamate excitotoxicity. The loss of dendritic spines, where Ng is mainly localized, may be associated with α7-nicotinic receptors via internalization of NMDAR and lead to impaired glutamatergic transmission [11,18,31]. Minor forms of Aβ may trigger the astrocytic release of glutamate and extrasynaptic NMDARs activation, which may promote the β-secretase processing of APP leading to increased Aβ production [32]. One potential explanation for these pathological processes may be synaptic depression and persistent dendritic loss dependent on Aβ [18]. Oligomers may also trigger dendritic pruning and toxicity, which could explain the sub-high concentration of Ng localized on dendritic spines [33]. However, Aβ oligomers also influenced the combined effects of impaired glutamate uptake and their excessive concentration in the presynaptic space, which increases the level of Ca2+ inside the neurons [34,35]. Given the above mechanisms, it seems advisable to test the Aβ42/Ng ratio. Our study demonstrated a significant diagnostic value of the Aβ42/Ng ratio in all compared groups. Interestingly, its usefulness in the differentiation of AD and MCI patients based on the AUC value seems to be better than other biomarkers, such as Aβ42/40, tTau, and pTau181. The relationship between Ng and amyloid may be significant for monitoring disease progression related to synaptic loss and disrupted transmission. 

The correlation between Ng and NPTXR in the MCI group appears to reflect mechanisms strongly related to impaired transmission of glutamatergic synapses but in different receptors. In the presence of excess glutamate induced by Aβo, the transmembrane domain of NPTXR is cleaved. Both NPTXR and AMPAR are internalized by endocytosis, which can be interpreted in the context of their early down-regulation. However, excitotoxicity may reflect the altered mechanism of decreased detection of glutamate and endocytosis of NPTXs family complexes and AMPARs. Moreover, the NPTXR/Ng ratio seems to be the most promising in differentiating MCI from CTRL, which is supported by the highest AUC score. The ratio of two novel biomarkers related to synaptic dysfunction gave better results than their separate analysis. The early changes and disruption of synaptic transmission, which also seem to be reflected in the above results, may also be related to Aβ oligomers [14,19].

### Future Directions and Challenges

Bioinformatics analyses are increasingly used to search for associations between protein-coding genes and their functions that may be significantly involved in neurodegeneration. Therefore, it seems reasonable to use enrichGo to search for similar functions of the tested proteins. Furthermore, this functional analysis based on MF expands the knowledge of potential protein interactions and common functions related to neuropathology. These approaches in biochemical research are not common but seem to carry additional knowledge about the tested proteins. However, any result indicating that a group of proteins or a pair of proteins is significantly enriched should be checked against available studies. Perhaps the biggest challenge is establishing the procedure and interpretation of enriched results in proteomic studies, especially about which background should be chosen. Performing GO enrichment analysis based on the whole genome or downregulated genes/proteins compared to upregulated genes/proteins can significantly affect enrichment results. Research on functional analysis and procedures or guidelines in proteomics should be continued and replicated by other researchers.

## 4. Materials and Methods

The study population involved *n* = 70 (*n* = 48 women, *n* = 24 men, 73 median years) subjects from the Department of Neurology, Jagiellonian University Hospital, Krakow, Poland, and included 33 AD patients (age: 76 (68–81)), 18 subjects with MCI (age: 75 (70–78)), and 19 non-demented controls (age: 66 (63–71)). In the clinical diagnosis of study groups, standard medical, physical, and neurological examination, laboratory screening tests, a comprehensive neurocognitive evaluation, and magnetic resonance imaging or computed tomography of the brain were used. Information on the past medical history of patients was also verified. Patients with alternations in CT or MRI suggesting cerebrovascular disorder and subjects with increased albumin quotient (QAlb) indicating blood-CSF barrier dysfunction were excluded from the study. The diagnosis of AD and MCI were based on the recommendations from the National Institute on Aging and Alzheimer’s Association (NIA-AA) criteria. Neuroimaging and neuropsychological examinations were combined with neurochemical findings (levels of Aβ1–42, Tau, and pTau181 and values of the Aβ1–42/Aβ1–40 ratio) for the most accurate clinical diagnosis of AD and MCI patients. The Erlangen Score algorithm was used for the interpretation of CSF biomarkers. The biochemical characteristics of study participants based on the concentrations of classical biomarkers for AD and CSF parameters are presented in Table 1. The MMSE score (range 0–30) was used to assess dementia severity (AD patients (MMSE: 22 [0–28]), MCI patients (MMSE: 26.5 [26–29]), and 19 non-demented controls (MMSE: 28 [25–30])).

The control group consisted of people who did not have subjective memory disorders that did not fulfill the MCI criteria or recurrent headaches. A careful examination of subjects in the control group, with detailed analyses of the CSF, allowed us to exclude the symptoms’ organic background. No control group subjects showed any significant alternations in the established biomarkers for AD (levels of Aβ1–42, Tau, and pTau181). These findings were confirmed by the Erlangen Score of 0 points in all 19 subjects of this group.

### 4.1. Biochemical Measurements

Samples of CSF were put into polypropylene tubes by a lumbar puncture at the L4/L5 or L3/L4 interspace. All the CSF samples were centrifuged, aliquoted, and frozen at −80 °C until analysis. Biochemical measurements of tested proteins (Ng, NPTXR, VILIP1) in CSF and AD biomarkers (Aβ1–42, Aβ1–40, Tau, and pTau181) in CSF were performed in the Department of Neurodegeneration Diagnostics, Medical University of Bialystok, Poland. The concentrations of neurogranin were assessed with commercially available quantitative bead-based immunoassay (MILLIPLEX MAP Human Neuroscience Magnetic Bead Panel 2, HNS2MAG-95K, Merck KGaA, Darmstadt, Germany). The concentrations of NPTXR were assessed with a commercially available RayBioHuman NPTXR ELISA kit (ELH-NPTXR; Ray Biotech, Norcross, GA, USA). The CSF samples were diluted 25-fold in PBS and tested in duplicates. Absorbance was read at 450 nm. The assay was performed following the manufacturer’s instructions. Washing steps were completed using Biotek 405LS. For readout, 96-well plates and a Luminex^®^ 100/200™ analyzer (Luminex Corporation, Austin, TX, USA) were used. Standards and samples were run in duplicates with a coefficient of variance (CV) <20%.

The concentrations of neurochemical dementia diagnostics (NDD) biomarkers were measured in CSF using IBL kits (Hamburg, Germany) for Aβ1–42 and Aβ1–40 and Fujirebio kits (Gent, Belgium) for t-Tau and pTau181 proteins.

### 4.2. Statistical Analysis

Statistical analysis was performed by nonparametric tests and analysis using the *PMCMRplus* package in the statistical software R RStudio: Integrated Development for R. RStudio (Version 1.2.5019), PBC, Boston, MA, USA. The Shapiro–Wilk test revealed that the concentrations of the tested proteins did not follow a normal distribution. The comparison between AD, MCI, and the control group was performed using the Kruskal–Wallis test. Subsequently, significant differences between the levels of the tested groups were analyzed using the post hoc Dwass Steele–Critchlow–Fligner test to verify in which groups the difference was statistically significant. The results are presented as medians and interquartile ranges, and statistical significance was set at *p* < 0.05. Additionally, the receiver operating characteristic (ROC) curve and area under curve (AUC) analysis were used to determine tested proteins’ diagnostic usefulness as candidate biomarkers. Gene Ontology (GO) enrichment analysis was performed using a Bioconductor package (ClusterProfiler). The whole genome was used as a background. 

## 5. Conclusions

The Ng, NPTXR, and the ratios of NPTXR/Ng, as well as Aβ42/Ng, were significantly different in the MCI patients compared to the CTRL group. Furthermore, the NPTXR/Ng ratio presented the highest diagnostic usefulness for differentiation of the above-mentioned groups, whereas the AUC for Aβ42/Ng ratio was high in all compared groups. The preliminary and screening bioinformatic analysis of pathways and functions based on enriched GO enabled a deeper understanding of the biological mechanisms of this disease. The combination of proteomic results and GO enrichment analysis seems particularly promising in generating new research objectives and possible therapeutic targets, and it seems that it is particularly important to apply and compare the results of empirical studies with bioinformatic analyses to better understand AD disease.

## Figures and Tables

**Table 1:** GO enrichment analysis for biological processes in terms of genes related to tested proteins in CSF.

ID	Description	GeneRatio	*p*-Value	p.Adjust	Q Value	Gene ID
GO:0050804	modulation of chemical synaptic transmission	4/5	<0.001	0.000247178	7.87172 × 10^−5^	APP/NRGN/MAPT/NPTXR
GO:0099177	regulation of trans-synaptic signaling	4/5	<0.001	0.000247178	7.87172 × 10^−5^	APP/NRGN/MAPT/NPTXR
GO:0048167	regulation of synaptic plasticity	3/5	<0.001	0.001265604	0.000403049	APP/NRGN/MAPT

**Table 2:** Biochemical characteristics of the study groups.

Tested Variables in CSF	Median (Range of Interquartile)			p (Kruskal–Wallis Test)	p (Dwass–Steele–Critchlow–Flinger Test)		
	AD	MCI	Controls		AD vs. CTRL	AD vs. MCI	MCI vs. CTRL
Tau (pg/mL)	671 (559–978)	389 (327–495)	220 (187–269)	<0.001	<0.001	<0.001	<0.001
pTau181 (pg/mL)	82 (68–113)	57 (47–68)	37 (33–41)	<0.001	0.001	<0.001	0.002
Aβ42/40 ratio	0.032 (0.02–0.04)	0.044 (0.03–0.06)	0.071 (0.06–0.08)	<0.001	<0.001	<0.001	0.006
Aβ42	500 (383–600)	802 (474–1045)	923 (804–1003)	<0.001	<0.001	0.012	0.833
NPTXR (pg/mL)	15 (11–18)	14 (10–15)	19 (16–21)	0.003	0.027	0.349	0.003
Ng (pg/mL)	869 (655–1171)	692 (499–833)	468 (419–560)	<0.001	<0.001	0.041	0.025
VILIP-1 (pg/mL)	0.109 (0.07–0.16)	0.09 (0.05–0.11)	0.036 (0.02–0.07)	<0.001	<0.001	0.269	0.04
Aβ42/Ng	53.9 (42–72)	117 (101–160)	191 (164–205)	<0.001	<0.001	<0.001	0.002
NPTXR/Ng	1.38 (1.17–2.18)	1.73 (1.58–2.36)	3.83 (3.62–4.31)	<0.001	<0.001	0.088	<0.001

**Table 3:** AUC of tested parameters in compared groups.

Tested Parameters	ROC Criteria in AD Compared to CTRL				ROC Criteria in MCI Compared to AD				ROC Criteria in MCI Compared to CTRL			
	AUC	SE	95% C.I. (AUC)	*p* (AUC = 0.5)	AUC	SE	95% C.I. (AUC)	*p* (AUC = 0.5)	AUC	SE	95% C.I. (AUC)	*p* (AUC = 0.5)
Ng	0.919	0.036	0.847–0.99	<0.001	0.707	0.074	0.562–0.852	0.005	0.749	0.084	0.583–0.914	0.003
NPTXR	0.716	0.07	0.578–0.854	0.001	0.589	0.083	0.433–0.762	0.121	0.813	0.076	0.665–0.961	<0.001
VILIP-1	0.805	0.064	0.679–0.93	<0.001	0.632	0.079	0.477–0.787	0.095	0.708	0.088	0.535–0.88	0.018
Aβ42	0.894	0.049	0.797–0.991	<0.001	0.736	0.078	0.582–0.89	0.002	0.556	0.103	0.353–0.758	0.590
Aβ42/40	0.952	0.047	0.861–1	<0.001	0.827	0.064	0.701–0.952	<0.001	0.800	0.075	0.653–0.946	<0.001
pTau181	0.986	0.012	0.962–1	<0.001	0.798	0.064	0.673–0.923	<0.001	0.870	0.060	0.755–0.987	<0.001
Tau	0.987	0.011	0.965–1	<0.001	0.858	0.057	0.746–0.968	<0.001	0.871	0.059	0.756–0.987	<0.001
Aβ42/Ng	0.982	0.014	0.955–1	<0.001	0.909	0.042	0.828–0.991	<0.001	0.830	0.069	0.695–0.965	<0.001
NPTXR/Ng	0.943	0.034	0.877–1	<0.001	0.646	0.077	0.496–0.797	0.055	0.974	0.027	0.921–1	<0.001

## Data Availability

The data presented in this study are available on request from the corresponding author. Key data are stated in the text.
